# PaTz groups for primary palliative care: reinventing cooperation between general practitioners and district nurses in palliative care: an evaluation study combining data from focus groups and a questionnaire

**DOI:** 10.1186/1471-2296-15-14

**Published:** 2014-01-20

**Authors:** Annicka GM van der Plas, Martijn Hagens, H Roeline W Pasman, Bart Schweitzer, Marij Duijsters, Bregje D Onwuteaka-Philipsen

**Affiliations:** 1 VU University medical centre, Department of Public and Occupational Health, EMGO + Institute for Health and Care Research, Centre of Expertise in Palliative Care, P.O. Box 7057, 1007 MB Amsterdam, The Netherlands; 2 1ste Lijn Amsterdam (ROS), P.O. Box 206, 1000 AE Amsterdam, The Netherlands

**Keywords:** Palliative care, End of life care, Primary health care, Interprofessional relations, General practitioner, Primary care nursing

## Abstract

**Background:**

PaTz (an acronym for ‘PAlliatieve Thuis Zorg’; palliative care at home) is an intervention to improve palliative care provision and strengthen the generalist knowledge of palliative care. In PaTz general practitioners and district nurses meet on a regular basis to identify patients with palliative care needs and to discuss care for these patients. This study explores experiences with regard to collaboration between general practitioners and district nurses, and perceived benefits of and barriers for implementation of PaTz.

**Methods:**

This study is conducted within the primary care setting. Participants were 24 general practitioners who filled in a questionnaire, and seven general practitioners, five district nurses and two palliative care consultants who attended one of two focus groups.

**Results:**

PaTz led to improved collaboration. Participants felt informational and emotional support from other PaTz participants. Also they felt that continuity of care was enhanced by PaTz. Practical recommendations for implementation were: meetings every 6 to 8 weeks, regular attendance from both general practitioners and district nurses, presence of a palliative care consultant, and a strong chairman.

**Conclusions:**

PaTz is successful in enhancing collaboration in primary palliative care and easy to implement. Participants felt it improved continuity of care and knowledge on palliative care. Further research is needed to investigate whether patient and carer outcomes improve.

## Background

Palliative care aims at improving the quality of life of patients and their families facing the problems associated with life-threatening illness [1]. In palliative care, interprofessional collaboration may play a decisive role in the ability to match care to the preferences of patients and their carers, for instance to arrange care so the patient can die at home. Patients consider good collaboration between their general practitioner (GP) and other professionals crucial to care at the end of life [2].

Collaboration between district nurses (DNs) and GPs, in the Netherlands the main providers of palliative care at home, is not always found to be satisfactory [3]. In other countries difficulties in primary care collaboration are also reported [4-6]. With corporate growth and shifting of tasks within home care organisations, the so called ‘home teams’ (local collaboration between nurses and GPs) disappeared [7]. Collaboration nowadays is hampered by time and financial constraints. GPs increasingly work part-time and the caseload is often high [8,9]. Within home care teams caseload is also high [10], and a home care team consists more and more of lower educated nurses [7] who may have more difficulty in communicating with a GP on advanced topics such as palliative care needs of their patients. Furthermore, ambiguity towards collaboration is difficult to overcome. One study showed that even when the advantages outweighed the disadvantages, the GPs sometimes struggled with a fear of losing control over the patient. The GP was the one person with the overview of the patient’s situation, and that was under threat as it was to be shared by the team [11].

The Gold Standards Framework (GSF) in the United Kingdom [12] has been shown to improve collaboration in palliative care in care homes [13] and primary care [14]. GSF can be implemented locally on a small scale and in direct relation to patient care, therefore it provided a good basis to tailor the program to the Dutch health care setting. The result, PaTz (an acronym for ‘PAlliatieve Thuis Zorg’; palliative care at home), was introduced in the Netherlands, and aims to improve palliative care provision and strengthen the generalist knowledge of palliative care. The main goals of PaTz are to support participants to: i) identify patients in the last year of life; ii) assess their needs, symptoms and preferences; and iii) plan care so patients receive care according to their wishes. Cornerstone of PaTz are the interprofessional meetings between GPs and DNs, with support from a palliative care consultant (physicians and nurses with formal training and experience in palliative care). Participation in (multidisciplinary) case discussions are associated with less perceived obstacles in delivery of primary palliative care [15]. Additional information on the implementation of PaTz within the context of the Dutch health care system can be found in Table 1.

**Table 1:** Implementation of PaTz within the context of primary care in the Netherlands

Primary care in the Netherlands	In the Netherlands, there are 16.6 million inhabitants. Each year, 77,000 people die of non-acute illnesses and 31% of these die at home [16]. More than half (55%) of Dutch GPs work part time [8]. About half (54%) of GPs work in a group practice, 28% works in a duo practice and 18% of GPs work in a solo practice. GPs see on average three to five palliative care patients a year [15]. Home care is offered by 248 home care organisations and a further 255 care- or nursing homes that also offer home care [17]. There is variation in the services offered by different home care organisations (for instance, some but not all offer domestic help). Experience with and knowledge of palliative care is not readily available in all home care organisations. DNs and home support workers who are confronted with end-of-life care see on average 10 palliative care patients a year [18].
Implementation of PaTz in the Netherlands	PaTz started as a pilot in Amsterdam in 2010, after the initiators successfully recruited participants for four PaTz groups within their network. In Amsterdam, the capital of the Netherlands, there are 779 810 inhabitants (in 2011). There are approximately 2306 inhabitants per 1 fte GP availability [19] and over 50 home care organisations [20] in Amsterdam. The first four PaTz groups started with each nine to ten GPs and two to three DNs; a total of 39 GPs from 18 practices (between one and four GPs per practice) and 10 DNs from four different home care organisations. Every two months 60 – 90 minute meetings are held. The meetings are prepared and chaired by one of the participants, mostly a GP, and a palliative care expert is present to provide information on palliative care when needed. During the meetings, patients with palliative care needs are identified and discussed. Also, a topic of interest can be discussed, like a new type of pain medication. During the meetings most of the time is used to discuss the specific needs of patients in palliative care and to organise that care. When needed thematic issues are more in full discussed with the assistance of the present expert. The chairpersons were trained before implementation by the PaTz initiators. The DNs are cleared by their organisations to attend PaTz meetings.

A study on the first four PaTz groups in the Netherlands was undertaken to provide insight into perceived consequences of PaTz on delivery of care, and offer practical guidelines for future implementation. This article examines 1) the experiences of GPs and DNs with regard to interprofessional cooperation, and 2) perceived benefits of and barriers for implementation of PaTz within the primary care setting.

## Methods

### Design and population

Data came from a questionnaire and focus groups. The questionnaires were sent to GPs one and a half year after start of PaTz. The 35 GPs involved in PaTz at that moment were invited to participate. Qualitative data came from two mixed (heterogeneous) focus groups, for which all participants of the first four PaTz groups were invited. The aim of the questionnaires was to gain insight in possible effects of PaTz. The focus groups were conducted to gather more in-depth information on how participants experienced PaTz to support future implementation of PaTz. Under Dutch law this study is exempt from approval from an ethics committee.

### Questionnaire

The questionnaire was sent to GPs who participated in the PaTz groups one and a half year after implementation of PaTz. Because of financial and time constraints no questionnaires were sent to the DNs. For the purpose of this article we used the information from the questionnaire that was relevant to either interprofessional cooperation or implementation of PaTz. Four questions regarding demographic information of the GP and five questions on PaTz were used. Three questions on PaTz were rated on a 5-point Likert scale (from ‘totally agree’ to ‘totally disagree’) regarding support, continuity of care and the presence of a consultant in palliative care. The fourth was an open ended question asking about the most important aspect of PaTz meetings. And the fifth question on whether the collaboration with the DN had improved could be answered with ‘yes’ or ‘no’.

### Focus groups

The two focus groups were conducted by a moderator (MH) and observer (AvdP). In preparation for the focus groups, the observer and moderator have been present during two PaTz meetings to familiarise themselves with the subject and with the structure of PaTz meetings. Firstly, participants were asked if they thought PaTz affected the care they gave. Secondly, participants were asked about points of improvement and strengths of PaTz meetings. The topic list for the focus groups was governed by the questionnaire, to offer more in-depth information on perceived consequences of PaTz. There was sufficient opportunity to bring up new topics and other experiences with PaTz within the focus groups. The focus groups were held on ‘neutral’ terrain (VU medical center) and took two hours. The groups were heterogeneous, but the last half hour was spent on separate group interviews for GPs and DNs to offer each discipline the chance to safely bring up additional information on interprofessional cooperation. The GP palliative care consultant joined the GPs and the DN palliative care consultant joined the DNs. Participants received a gift certificate of 20 euros.

### Data analysis

For the questionnaire frequencies were calculated. The focus groups were recorded, transcribed and a thematic analysis was conducted. The following themes were explored:

cooperation between the GPs and DNs before and after implementation of PaTz,

perceived benefits of participating in PaTz, successful attributes of PaTz,

perceived barriers to participating in PaTz, unsuccessful attributes of PaTz.

The perceived benefits and barriers were literally asked about in the focus groups, these themes were explored with future implementation guidelines in mind. The theme of cooperation was included in the topic list based on theoretical expectations. Data were re-arranged on the themes by A.v.d.P., and this arrangement and also possible interpretations, associations and explanations were then discussed with M.H., H.R.W.P. and B.D.O-P.

## Results

### Respondents

The questionnaire was sent to 35 GPs in April 2011. Four questionnaires came back because the GP no longer held practice, another was excluded because that GP had not participated in the PaTz meetings. The questionnaire was filled in by 24 GPs (80% of 30); 14 women and 10 men with a mean age of 51 years. Most of the GPs (75%) worked part time, and 17 GPs (81.0%) had had some kind of training in palliative care.

The focus groups were attended by seven GPs (3 men, 4 women), five DNs (2 men, 3 women) and two palliative care consultants (1 male GP, 1 female nurse). Participants were from three different PaTz groups. The quotations below come from 6 different participants (3 GPs and 3 DNs).

#### 1) Cooperation

One of the most mentioned pros of PaTz was the renewed cooperation between the GP and DN. This was brought forward by 9 out of 24 respondents in the open question in the questionnaire (Table 2) and 20 out of 24 respondents answered ‘yes’ in the question whether collaboration had improved with implementation of PaTz. Improved collaboration was a leading theme in both focus groups. Because of PaTz the two disciplines discussed patients together and learned to work together. A direct result of this renewed cooperation can be seen in the support participants of PaTz experienced (e.g. sharing and appreciating each others expertise) and also in continuity of care (e.g. a lower threshold to contact each other).

**Table 2:** Opinions on PATz as expressed in a questionnaire filled in after implementation

	**n (n** = **24)**	**%**
For you, what is the most important aspect of PaTz meetings? (open question, more than one answer possible)	(n = 23; 1 missing)	
- Support from others: sharing experiences and/or discussing problems and solutions	16	69.6%
- (Reinventing) cooperation: better cooperation between GP and DN, knowing each other	9	39.1%
- Continuity of care: better overview of patients and what needs to be done for patients, better/more proactive care	6	26.1%
PaTz supports me in caring for patients with palliative care needs		
- Totally agree + agree	17	70.8%
- Neutral	5	20.8%
- Disagree + totally disagree	2	8.3%
PaTz enhances the continuity of care for patients with palliative care needs		
- Totally agree + agree	13	54.2%
- Neutral	9	37.5%
- Disagree + totally disagree	2	8.3%
Presence of a consultant (expert) in palliative care is useful in PaTz meetings		
- Totally agree + agree	21	87.5%
- Neutral	2	8.3%
- Disagree + totally disagree	1	4.2%
Collaboration with the DN is improved by PaTz, answer yes	20	83.3%

##### 1a) Support

In the questionnaire, the statement ‘PaTz supports me in caring for patients with palliative care needs’ was received with (total) agreement for 17 of 24 respondents (Table 2). In the focus groups support was highlighted in several ways. Firstly, informational support in offering care for patients with palliative care needs was felt because knowledge on diseases, symptoms, and medication was shared. This happened within the professions and between the GPs and DNs. According to participants this had a direct positive effect on patient care but also on views and opinions of colleagues and between disciplines. Participants saw each other as more equal partners and appreciated each other’s expertise more than before participating in PaTz:

“I find it good to have discussions with the DNs because they cover a different aspect of healthcare. … I find that adds something to my own view of things. A different perspective, from a different background. Everyone has their own area of expertise. This makes the best possible use of that”. (GP, Focus group 1)

Second, emotional support was felt because a safe environment was created to discuss what is difficult in palliative care, and what went wrong and right in caring for patients:

“I find the work has got less lonely. I think it’s great that at least now you and the nurse are worrying about things together. It helps improve our quality. You don’t have that ‘lonely at the top’ feeling any more”. (GP, Focus group 1)

##### 1b) Continuity of care

The statement ‘PaTz enhances the continuity of care for patients with palliative care needs’ was met with (total) agreement for 13 out of 24 respondents (Table 2). In the focus groups participants indicated that continuity of care was enhanced in several ways. Firstly, most mentioned, the communication between GPs and DNs had improved. There was better use of each other’s expertise. Because the GP and DN worked as a team, time was spend more efficiently:

“You save time, because you don’t need to call the GP a hundred times before you can ask a question – when you call, the GP knows that you call with good reason. That saves time. And also the other way around, the GP can call you instead of visiting the patient for a second time that day”. (DN, Focus group 2)

Contact between the DN and other nurses in their home care organisation was also important in that respect; the DN that participated in PaTz functioned as an intermediary for communication with other professionals in the home care organisation. Contact was established earlier in the course of disease of the patient and there was more contact. An important tool for introducing home care timely to patients are continuity visits (a home visit by a DN to offer information on possibilities of home care, this visit is free of charge for the patient), but most GPs did not know this existed before PaTz:

“You get the home care service involved at an earlier stage. Because you think ‘that’s going to go wrong in three to six months’ time’ and you can already start thinking about the continuity visits from the DN. I didn’t even know they existed before. And then you’re more likely to set up a small team for the patient and you get a better picture of the patient, even if you haven’t seen them yourself for a while”. (GP, Focus group 1)

It was felt that the contact between the GP and DN was also important for patients, that it made patients more secure about the care they were given.

Secondly, some GPs were more aware of the need for clear communication with the out-of-hours locums. However, other GPs noted that there was still some room for improvement in this regard. Thirdly, because of PaTz, care was experienced as more comprehensive and more pro active, which are important aspects in continuity of palliative care.

#### 2) Practical recommendations for implementation

In the focus groups the participants mentioned some practical recommendations they thought important for successful PaTz groups. The optimal frequency for PaTz meetings appeared to be around 6 times a year, groups aimed at meeting once every 6 to 8 weeks (taking into account holidays and other reasons for cancellation). However, for the GPs not working in a primary care centre or in an arrangement of co-location with other GPs, the time between meetings could be long and follow up on problems that were discussed during a meeting, was felt to be missing.

There must be a right mix of GPs and DNs present during meetings, for the interprofessional assets of PaTz to come about. DNs should have affinity with and experience in palliative care and employed by the home care organisations the GPs most commonly work with. For DNs it was important to make their mark during the first few meetings; if they actively engaged in discussions and brought forward topics, this helped in becoming equal partners in meetings. Being outnumbered by GPs in the PaTz meetings made it difficult to do this:

“We [nurses] need to project a clear image and I think we’ve come a long way in that respect. And I have the impression that they [GPs] have started treating us much more as equals as a result. But you still notice how deeply rooted the ‘us versus them’ culture is”.

“It was the same in our group, the doctors took the initiative and they worked together really well, and the first three times I was wondering ‘what am I doing here actually?’ … But you gradually saw a change, a trust and you were able to make your points”. (Exchange between two DN’s, Focus group 2)

Participants should be committed to coming to the meetings, so continuity of persons is safeguarded. The chairman should make sure meetings are constructive and well-arranged. Also, an expert on palliative care should be there. This is also expressed in the questionnaire, in which 21 of 24 respondents agreed or totally agreed with the statement ‘Presence of a consultant (expert) in palliative care is useful in PaTz meetings’ (Table 2).

Finally, PaTz was felt to be a simple but efficient method in improving palliative care and interprofessional collaboration, which stimulated the presence of participants, but adding tools and guidelines to the PaTz method would bare the risk of making it less attractive to work with. This was especially mentioned by GPs, and in both focus groups.

## Discussion

This study shows that PaTz is easy to implement and is successful in bringing together primary care teams. This renewed cooperation led to informational and emotional support being felt in PaTz meetings and in the perception of participants improved continuity of care for patients with palliative care needs. PaTz meetings must be attended by the right mix of committed GPs and DNs. A consultant in palliative care has to be present. According to GPs, a risk for PaTz is when participants become too ambitious in embellishing it with additional tools and guidelines. There is tension between the ease of implementation of PaTz and further improving the quality of palliative care by extending PaTz.

### Strengths and weaknesses of the study

This study provides valuable information on how participants experience PaTz. This can guide future implementation of PaTz and thereby improve generalist palliative care. In the Netherlands, PaTz is the first intervention set up to improve cooperation between GPs and DNs. Also, it demonstrates that GSF can be successfully adapted to health care settings outside the United Kingdom. Although participants stated that quality of care had improved, another study is necessary to assess effect on patient outcomes. Another limitation is the limited number of participants in this study, because this study was done with the first four PaTz teams. Results may not be representative for teams that are less willing to implement PaTz. This study was conducted within the Dutch health care system, with GPs and DNs as the main providers of palliative care at home. The Dutch GP has a function as gate keeper and refers the patient to home care when necessary. In other health care systems collaboration and interdependencies between the GP and DN may be different, for instance because of the existence of specialised palliative care teams in primary care. Also, the reward of a gift certificate could have enticed the participants of the focus groups to bring forward mostly positive aspects of PaTz. However, this is unlikely to have happened since the participants actively brought forward difficulties and problems with palliative care provision and doubts on aspects of PaTz.

### Successful interprofessional cooperation

The PaTz groups performed well according to respondents; participants were enthusiastic about the meetings and committed to them. This is in line with Petrie’s framework for interprofessional work [21]. Petrie considers four factors of importance to success of interprofessional cooperation; idea dominance, psychological characteristics of participants, institutional setting, cognitive maps.

### Idea dominance

Idea dominance relates to a clear and recognisable idea that serves as a central focus, with feedback to measure or express achievements. The aims of PaTz, to improve palliative care provision and strengthen the generalist knowledge of palliative care, were made clear to all participants at start of implementation. Attendance was generally high because participants felt the meetings were useful in meeting these aims. They became more aware of their own role in improving palliative care (e.g. with regard to sharing information), gained knowledge on palliative care, received practical advise from others, and felt they were actually discussing issues that really matter and are central to quality of care. Similarly, in a study on the implementation of GSF in primary care trusts in the United Kingdom, it was seen that recognition of the value of GSF for interprofessional cooperation and to improve care provision, was a driver for adoption of GSF [22].

### Psychological characteristics of participants

The second factor in Petrie’s framework is ‘psychological characteristics of participants’: participants must be secure in their own competencies, have a taste for adventure, have broad interests, must be willing to learn from each other, must feel that they achieve something on a personal level. Within PaTz, participants were willing to invest in each other, they got to know each other by working together and discussing patients face to face. Authority issues and communication problems were overcome during the first year. Participants worked through these; they did not stop the meetings. In Tuckman’s model of group development [23] four stages (forming, storming, norming, and performing) are distinguished; it seems that the PaTz groups were all in the stage of performing during the focus group study. Reluctance of GPs to share information on their patient and lack of responsiveness of GPs, as reported elsewhere by DNs [4] were diminished by the PaTz meetings. The finding that initiatives such as PaTz have a high impact on interprofessional working can also be seen in a review study on GSF [12] in which this is also a prominent and repeated finding.

### Institutional setting

The third factor of importance mentioned by Petrie is the institutional setting; administrative support, peer recognition from outside or from within the group itself. In the aforementioned review of GSF [12] low performance within GSF was associated with conflicting organizational priorities. This was no issue in this study, since the GPs and DNs readily agreed to participate in PaTz. All participants were supported and facilitated by their organisation to attend meetings.

Commitment and stability in group membership is seen as an important asset. It creates a safe environment to discuss what is difficult in providing care for patients and exchange knowledge. Also, by working more closely together with the DN in daily care for a patient the GP feels support, the observation of one GP that her work ‘is less lonely’ is an example of this. This is also seen with GPs in other interprofessional groups [11].

### Cognitive maps

Cognitive maps refer to basic concepts and general ideals. Petrie states that cognitive maps of participants must be recognised and shared. To do this tacit knowledge must be made explicit. People from different disciplines may look at the same and observe different things. Also, they may have different meanings for the same terms. GPs and DNs in the Netherlands are generally used to communicate in writing in a dossier at the patients’ house were both the GP and DN record main activities, important appointments and agreements. But communication in writing may hamper the understanding of each other’s cognitive maps. Indeed, notes from the GP are not always useful to guide nursing practice [24] and vice versa the GP is overwhelmed by information given by nurses [25]. Within discussions of individual patients during PaTz meetings, more insight may occur into the cognitive maps; tacit knowledge existing within the professions becomes shared knowledge, applicable also to other patients than those discussed during PaTz meetings.

## Conclusion

PaTz is successful in bringing back primary care teams for patients with palliative care needs. Although palliative care in itself already has an interdisciplinary work focus, PaTz was needed to help GPs and DNs to bring about cooperation. This reinvention of cooperation leads to better continuity of care as perceived by participants, more knowledge on palliative care and the patient, and support in difficult situations regarding care, as experienced by the participants of PaTz. Implementation of PaTz is straightforward; it takes 6 meetings per year, a well prepared and strong chairman, about 8 to 12 GPs, 3 DNs with experience in palliative care, and a palliative care consultant.

## Abbreviations

PaTz: *P*alltiatieve *T*huis *Z*org; palliative care at home; GSF: Gold standards framework; DN: District nurse; GP: General practitioner.

## Competing interests

The authors declare that they have no conflict of interest.

## Authors’ contributions

AvdP participated in the design of the study, was observer in the focus groups, analysed and interpreted the data, and drafted the manuscript. BO-P conceived of the study, carried out the measurements, participated in its design and coordination and made substantial contributions to the data interpretation and writing of the paper. MH prepared and was moderator of the focus groups, analysed and interpreted the data from the focus groups, and participated in critical revision of the manuscript for important intellectual content. HRWP carried out the measurements, participated in its design and coordination and made contributions to the data interpretation and writing of the paper. BS and MD participated in design of the study, interpretation of the data and critical revision of the manuscript for important intellectual content. All authors read and approved the final manuscript.

## Authors’ information

Three of the authors (BS, MD, BO-P) are members of PaTz Foundation, which was established in the last trimester of 2012 to further develop PaTz through evaluation and to facilitate implementation of PaTz in the Netherlands.

## Pre-publication history

The pre-publication history for this paper can be accessed here:

http://www.biomedcentral.com/1471-2296/15/14/prepub
